# Antifungal Activity of Disalt of Epipyrone A from *Epicoccum nigrum* Likely via Disrupted Fatty Acid Elongation and Sphingolipid Biosynthesis

**DOI:** 10.3390/jof10090597

**Published:** 2024-08-23

**Authors:** Alex J. Lee, Joseph Hammond, Jeffrey Sheridan, Simon Swift, Andrew B. Munkacsi, Silas G. Villas-Boas

**Affiliations:** 1 School of Biological Sciences, University of Auckland, Auckland 1010, New Zealand; alexleenz@hotmail.com; 2 School of Biological Sciences, Victoria University of Wellington, P.O. Box 600, Wellington 6140, New Zealand; joe.hammond1895@gmail.com (J.H.); jpcsheridan@hotmail.com (J.S.); 3 Faculty of Medical and Health Sciences, University of Auckland, Auckland 1023, New Zealand; s.swift@auckland.ac.nz; 4 Luxembourg Institute of Science and Technology, Environmental Research and Innovation Department, L-4362 Esch-sur-Alzette, Luxembourg

**Keywords:** *Saccharomyces cerevisiae*, mechanism of action, polyene, fungicide, sphingolipid, elo2, metabolomics

## Abstract

Multidrug-resistant fungal pathogens and antifungal drug toxicity have challenged our current ability to fight fungal infections. Therefore, there is a strong global demand for novel antifungal molecules with the distinct mode of action and specificity to service the medical and agricultural sectors. Polyenes are a class of antifungal drugs with the broadest spectrum of activity among the current antifungal drugs. Epipyrone A, a water-soluble antifungal molecule with a unique, linear polyene structure, was isolated from the fungus *Epiccocum nigrum*. Since small changes in a compound structure can significantly alter its cell target and mode of action, we present here a study on the antifungal mode of action of the disalt of epipyrone A (DEA) using chemical-genetic profiling, fluorescence microscopy, and metabolomics. Our results suggest the disruption of sphingolipid/fatty acid biosynthesis to be the primary mode of action of DEA, followed by the intracellular accumulation of toxic phenolic compounds, in particular *p*-toluic acid (4-methylbenzoic acid). Although membrane ergosterol is known to be the main cell target for polyene antifungal drugs, we found little evidence to support that is the case for DEA. Sphingolipids, on the other hand, are known for their important roles in fungal cell physiology, and their biosynthesis has been recognized as a potential fungal-specific cell target for the development of new antifungal drugs.

## 1. Introduction

Polyenes, azoles, allylamines, echinocandins, and flucytosines are the five major classes of antifungal drugs that are currently available on the market to combat fungal diseases with distinct modes of action [1,2,3]. Even though these antifungal drugs are currently used widely to treat an increasing number of fungal infections in a variety of hosts, two major limitations are present: (i) fungal strains are resistant to their antifungal modes of action [4,5], and (ii) their toxicity to the hosts (e.g., mammalian cells) due to their lack of selectivity towards the fungal target [6,7]. To overcome these limitations, increasing the effectiveness while decreasing the toxicity of the current antifungal drugs was attempted through the synthetic modification of the chemical scaffolds of existing antifungal drugs [8,9] and improved delivery systems of the antifungal drugs [10]. However, the antifungal modes of action of those improved drugs remains the same [10,11]. Since an antifungal compound can still be active against fungal strains that are resistant to other antifungal modes of action [12,13], the discovery of new compounds with novel antifungal modes of action and higher selectivity towards the target could be the starting point to address the limitations of the currently used antifungal drugs.

Natural products derived from microorganisms, including fungi, are already well-known as great sources of bioactive compounds with various biological activity [4,9,14,15,16]. *Epicoccum* species (Fungi, Ascomycota) are reported to produce several biologically active metabolites such as the antibacterial flavipin (3,4,5-trihydroxy-6-methyl-O-phthalaldehyde) [17,18], as well as epicoccamides and thiornicin that show anti-tumour properties [14,19]. Epipyrone A is a secondary metabolite with antiviral and telomerase inhibitory activity that was previously isolated from an unidentified species of *Epicoccum* [20,21]. Previously, we reported the purification, chemical properties, structure, and antifungal activity of the disalt of epipyrone A (DEA) (presumably di-sodium based on the mass spectrometry results) produced by the fungus *Epicoccum nigrum* ICMP 19927 [22]. The non-salt form of epipyrone A shows low antifungal activity probably due to its poorer solubility in water. Interestingly, DEA showed a linear polyene structure unlike any other antifungal drugs of the same family, such as amphotericin B and nystatin, which are cyclic. It also possessed a glycosylated α-pyrone moiety, which is rare in nature and increases its water solubility [20,23]. A minor change in the chemical structure of a compound can have significant effects on its biological activity and mode of action [13,23,24,25,26]. In addition, the polyene class of antifungal compounds is of current interest due to its broad antifungal activity spectrum [3,27]. Here, the antifungal mode of action of DEA was investigated via chemical-genetic profiling, fluorescence microscopy, and metabolomics. We observed *Saccharomyces cerevisiae* sphingolipid biosynthesis via fatty acid elongation as a potential target for the antifungal activity of DEA, which was followed by a drastic increase in the levels of toxic phenolic acids inside the fungal cells, primarily *p*-toluic acid (4-methylbenzoic acid).

## 2. Materials and Methods

### 2.1. Chemicals

All chemicals used in this study were of analytical grade. Methanol, chloroform, sodium bicarbonate, and sodium hydroxide were obtained from Merck (Darmstadt, Germany). The internal standard L-alanine-2,3,3,3-d_4_, the derivatization reagent methyl chloroformate (MCF), pyridine, D-glucose, and glycerol were purchased from Sigma-Aldrich (St. Louis, MO, USA). Anhydrous sodium sulfate was obtained from Fluka (Steinheim, Germany).

### 2.2. Fungal Strains

*Epicoccum nigrum* ICMP 19927, currently deposited at the International Collection of Microorganisms from Plants (https://www.landcareresearch.co.nz/ [accessed on 2 February 2016]), was used for the production of disalt of epipyrone A. *S. cerevisiae* strains in the haploid BY4741 background (MAT a his3∆1, leu2∆0, met15∆0, ura3∆0) were used in this study. *S. cerevisiae* strains were grown either on the YPD agar containing, in g/L, yeast extract (6), peptone (3), dextrose (10), and agar (15) at pH 5.5, or on the synthetic complete (SC) media buffered with MOPS (3-[N-morpholino] propanesulfonic acid). *E. nigrum* was cultured on Czapek Yeast Agar (CYA) medium containing, in g/L, sucrose (30), yeast extract (5), NaNO_3_ (6), K_2_HPO_4_ (1), KCl (0.5), MgSO_4_·7H_2_O (0.5), FeSO_4_·7H_2_O (0.01), and agar (15) at pH 6. The culture plates were incubated at 25 °C in the dark for 14 days.

### 2.3. Extraction and Purification of Disalt of Epipyrone A (DEA)

Under low light intensity, 20 plates of 14-day-old *E. nigrum* cultures were first macerated into a 1L glass bottle by passing them through a 50 mL syringe (Terumo, Tokyo, Japan). The macerated agar + mycelia mixture was resuspended into approximately 400 mL of cold analytical-grade methanol (−20 °C), then the mixture was shaken at 180 rpm for 1 h, at 4 °C, and protected from light. The fungal biomass and agar debris were removed from the methanol extract through centrifugation at 4000 rpm for 30 min, at 4 °C, followed by vacuum-aided filtration using Whatman No. 1 filter paper. The purity of the crude methanol extract was confirmed using a semi-preparative HPLC, as previously described [22]. The filtered crude extracts were stored at −80 °C until further experiments.

The DEA was purified, as previously described [22]. In short, silica-gel column chromatography with LiChroprep RP-8 (40–63 µm, Merck) was used. Two volumes of dH_2_O were used to prime the column, then one volume of crude extract diluted with dH_2_O (1:5 *v*/*v*) was loaded. Two volumes of dH_2_O were used to wash and remove the sugar, then increments of dH_2_O and MeOH were applied to elute the orange fraction at 40–60% MeOH. This fraction was collected and dried under reduced pressure and freeze-dried. The product, orange powder, was stored at −20 °C until further experiments.

### 2.4. Liquid Bioactivity Assay

Ten different concentrations of disalt of epipyrone A (5, 10, 20, 25, 30, 35, 40, 45, 50, and 60 μg/mL) in SC broth were tested against the parental yeast strains. Honeycomb plates (Oy growth curves Ab Ltd., Helsinki, Finland) were used to grow the yeast, which was analysed with Bioscreen C (Oy Growth Curves Ab Ltd., Helsinki, Finland). This method was used to identify the IC_20_ (20% inhibitory concentration) and IC50 (50% inhibitory concentration) of DEA against the parental strains of *S. cerevisiae* strains, BY4741. Each well was inoculated with 1–5 × 10^5^ CFU/mL of yeast cells, and then it was incubated for 24 h at 30 °C in the dark. Yeast growth in the presence of different concentrations of the DEA was compared against the growth without the antifungal compound.

### 2.5. Chemical-Genetic Analysis of Haploid Deletion Library

Chemical-genetic screening was carried out using a haploid deletion library of *S. cerevisiae* consisting of approximately 4800 non-essential deletion mutant strains in 384 colony formats over 14 Singer plates (Singer Instruments, Roadwater, UK). These were replicated into 14 new Singer plates with SC medium in 1536 format to test each strain in quadruplicate, and then incubated overnight at 30 °C. Each of the plates (1–14) was arrayed onto SC agar and SC agar containing a semi-inhibitory concentration (50 μg/mL) of DEA. After 24 h of incubation at 30 °C in the dark, images of the cultured plates containing DEA were taken and compared against the corresponding control plates without the antifungal compound. These were processed with the R and “Gitter” package (version R 3.4.3) [28]. The mutant strains that showed changes in colony size and circularity (potential growth defect) on the test plates compared to the control were selected for further confirmation to eliminate false positives. Therefore, each selected mutant strain (in triplicate) was incubated in a 96-well plate containing 200 μL SC medium and DEA at 25.5 μg/mL (IC_20_). Each well was inoculated with 1–5 × 10^5^ yeast cells, and the plate was incubated at 30 °C for 14 h. The growth of the yeast strains was continuously monitored by a measurement of culture optical density at 600 nm using a Bioscreen C (Oy Growth Curves Ab Ltd., Helsinki, Finland).

### 2.6. Culture Preparation for Metabolomics 

Yeast strains grown on the YPD agar for 48 h at 28 °C were used to prepare the pre-inocula. A single yeast colony was inoculated into 500 mL flat-bottom flasks containing 200 mL SC medium, which were incubated at 30 °C and 180 rpm until reaching the mid-log growth phase. These were used to inoculate six new flasks containing 200 mL of SC medium at an initial OD600 of 0.1. The flasks were incubated at 30 °C and 180 rpm until reaching the log growth phase, when the first sampling for metabolome analysis was carried out (TB). Then, purified DEA dissolved in SC medium was added to three culture flasks of each strain and to one uninoculated control flask. The final concentration of DEA was set to 25.5 μg/mL for the BY4741 parental strain and the elo2∆ strain, which corresponded to their respective IC_20_. The remaining flasks were supplemented with the same volume of sterile blank medium without DEA. Each flask was sampled rapidly (within approx. 2 min) after the introduction of DEA (T0) and at the following time points: 1 h (T1), 2 h (T2), 3 h (T3), and 4 h (T4). The cultures were incubated in the dark to prevent the light degradation of the DEA.

### 2.7. Sampling and Extraction Procedure for Intracellular Metabolite Analysis

The sampling, quenching, and intracellular metabolite extraction were based on our previously published protocol [29]. In summary, 10 mL of culture broth were harvested and quenched from the culture flasks by rapidly mixing the samples with cold glycerol-saline solution (3:2), followed by centrifugation at −20 °C. The cell pellets were resuspended in cold glycerol–saline washing solution (1:1), followed by centrifugation at −20 °C. Intracellular metabolites were extracted from the cell pellets after the addition of cold methanol–water solution (1:1) at −30 °C and the internal standard (2,3,3,3-d_4_-alanine), followed by three freeze–thaw cycles. All extraction steps were carried out at −20 °C or below. The samples containing extracted intracellular metabolites were freeze-dried using a VirTis freeze-dryer from SP Scientific (Newtown Square, PA, USA). 

### 2.8. Extracellular Metabolite Analysis

Culture broth (10 mL) was harvested from the culture flasks and immediately filtered using a membrane filter (0.2 µm pore size). The filtrate was then separated into 3 aliquots (2 mL), and 20 µL of the internal standard (2,3,3,3-d_4_-alanine 10 mM) was added to each of them. The samples were frozen and subsequently freeze-dried on a VirTis freeze-dryer (SP Scientific, Newtown Square, PA, USA).

### 2.9. Separation, Identification, and Analysis of Metabolites

The freeze-dried intracellular and extracellular samples were resuspended using 200 mL of NaOH (1 M) and derivatized using the methyl chloroformate (MCF) method, according to our standard laboratory procedure described previously [29]. The derivatized samples were analysed by GC–MS, according to the parameters established previously [29], using a GC7890 gas chromatograph (Agilent Technologies, Santa Clara, CA), coupled with an MSD5975 mass spectrometer (Agilent Technologies, Santa Clara, CA, USA). AMDIS software (version 2.71) was used for deconvoluting the GC–MS chromatograms and identifying metabolites using a n in-house MCF mass-spectra library. The identifications were based on both the MS spectrum of the derivatized metabolite and its respective chromatographic retention time. The relative abundance of the identified metabolites was determined by ChemStation (Agilent Technologies, Santa Clara, CA, USA) by using the GC base–peak value of a selected reference ion. These values were normalized by the biomass content in each sample, as well as by the abundance of the internal standard (2,3,3,3-d_4_-alanine). A student’s *t*-test was applied to determine whether the relative abundance of each identified metabolite was significantly different between experimental conditions. The entire data mining and data normalization were automated in R software version 4.3.2, as described previously [29].

### 2.10. Proteome-Wide Protein Abundance and Localization Analysis

The yeast strains used for this experiment were derivatives of BY4741 [30]. The GFP yeast library consisted of 11 plates containing approximately 4100 strains. The abundance and localization of the proteins in the yeast proteome were measured using fluorescent microscopy. The 4100 strains comprising the GFP library were pinned into CellCarrier-384 Ultra Microplates (Perkin Elmer) clear-bottom tissue culture plates with either 30 μL of SC medium or 30 μL of 25.5 μg/mL DEA. The optical plates were incubated for 8 h at 30 °C. After incubation, each plate was imaged using an Incell analyser 6500HS (General Electric) using specific settings (Supplementary Table S1). Genes in which product localisation or abundance changed in response to DEA were analysed using the Gene Ontology term finder (https://go.princeton.edu/cgi-bin/GOTermFinder [accessed on 3 March 2021]) with Bonferroni correlation (*p* < 0.01) and all evidence codes.

### 2.11. Thin-Layer Chromatography (TLC)

Neutral lipids were visualized using thin-layer chromatography (TLC), as previously described [31]. Strains were grown overnight in SC medium at 30 °C with constant rotation. The cells were then diluted to a concentration of 2.55 × 10^6^ cells/mL in 7 mL of SC media and grown for 8 h in the presence or absence of DEA (25.5 μg/mL). Lipids were extracted using a Folch extraction [32] and separated using hexane/petroleum, ether/diethyl, and ether/acetic acid (50:20:5:1, *v*/*v*/*v*/*v*) as the solvent. The neutral lipids were stained with iodine and imaged using a digital camera.

## 3. Results

### 3.1. Bioativity of DEA in Yeast

**Figure 1:**
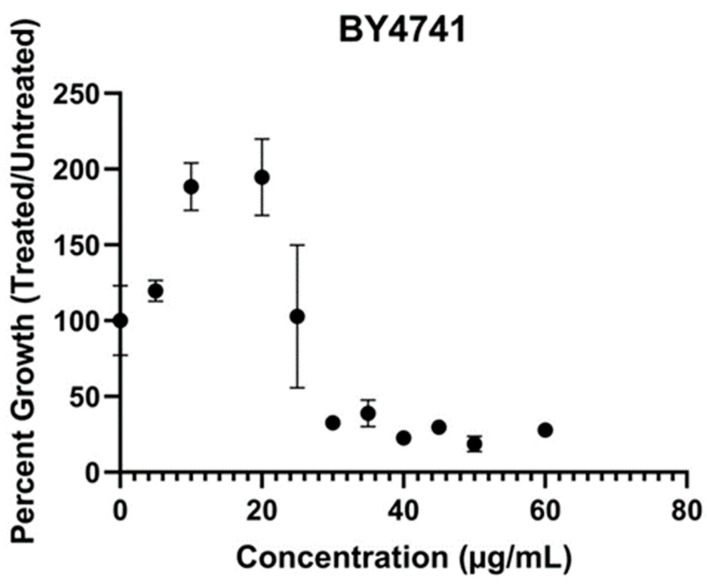
Growth of *Saccharomyces cerevisiae* BY4741 parental strain in presence of different concentration of disalt of epipyrone A (DEA). Cells were grown in absence or presence of DEA in synthetic complete broth. The growth was measured at the mid-exponential phase (14 h) with Bioscreen C at wavelength of 600 nm. The error bars show the standard deviation between technical replicates (*n* = 3).

Previously, we demonstrated the antifungal activity of DEA against several yeast and filamentous fungi [22]. The *S. cerevisiae* parental strain BY4741 was used to elucidate the mechanism of action of antifungal compounds. To better characterize the antifungal activity of DEA against *S. cerevisiae*, growth was measured in liquid cultures in the presence of varying concentrations of DEA (5–60 μg/mL). Relative to the control media, growth was increased by ~100% at 20 μg/mL DEA and inhibited by 20% at a slightly higher concentration (25.5 μg/mL DEA) (Figure 1). These results suggest the antifungal response of S. cerevisiae to DEA may be occurring in a pattern of hormesis, in which the concentration of the toxic threshold of DEA resulted in greater growth/adaptive response in the yeast.

### 3.2. Chemical-Genetic Profiling Distinguishes the Importance of Endocytosis, Fatty Acid Elongation, Cell Wall Integrity, and Actin Cytoskeleton as Mechanisms Buffering DEA Bioactivity

**Figure 2:**
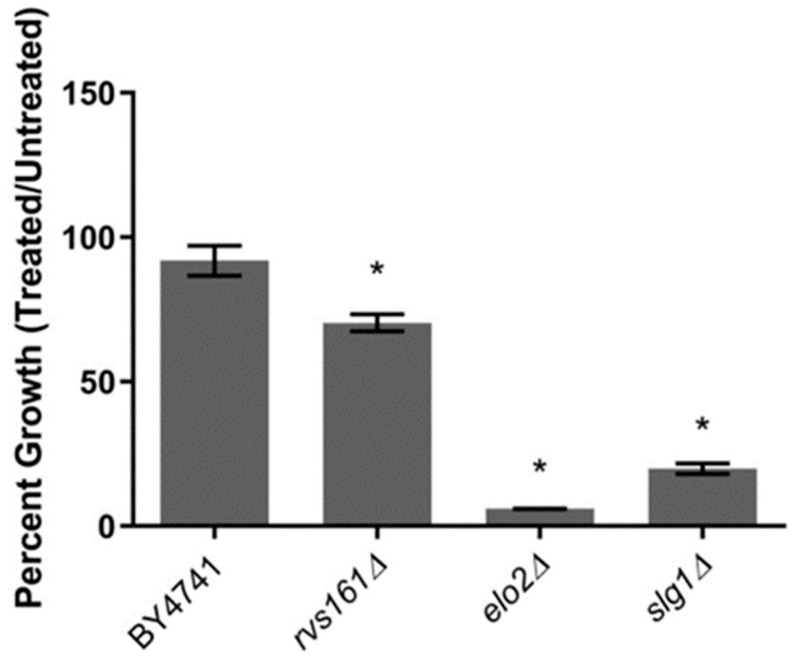
Hypersensitivity of three *Saccharomyces cerevisiae* deletion strains to the disalt of epipyrone A (DEA). Cells were grown in absence or presence of 25.5 µg/mL DEA (IC_20_ of the parental strain) in synthetic complete broth. The growth was measured at the mid-exponential phase of parental strain (14 h) with Bioscreen C at wavelength of 600 nm. The error bars show the standard deviation between technical replicates (*n* = 3). *, *p* < 0.05, student’s *t*-test comparing each deletion strain to BY4741.

Agar-based chemical-genetic analyses using an *S. cerevisiae* deletion collection have been used to determine the mechanism of action of hundreds of compounds [33]. To identify genes and their associated processes that buffer the mechanism of DEA, growth was quantified for ~4600 gene deletion strains on synthetic complete (SC) agar in the presence and absence of 50 μg/mL of DEA. This concentration inhibited the growth of BY4741 (wild type) yeast on agar by 20% compared to the untreated media, thus leaving open a large window (80%) to detect additional growth defects due to gene deletions. In high-throughput format (i.e., 384-colony format in quadruplicate), a significant growth defect was observed for 137 deletion strains (~3% of tested strains). To confirm that these knockout strains were truly sensitive, growth was measured in low-throughput format in liquid. Each strain was cultured in SC broth containing DEA at 25.5 μg/mL, an IC_20_ concentration in the parental BY4741 strain. Out of the 137 deletion strains, only 3 (rvs161∆, elo2∆, and slg1∆) exhibited statistically significant growth reductions (*p* < 0.05) in DEA compared to the control media (Figure 2). The most sensitive was elo2∆ (<10% growth), followed by slg1∆ (20% growth) and rvs161∆ (72% growth). RVS161 encodes a lipid raft protein in the cell wall that regulates the polarization of the actin cytoskeleton and endocytosis [34]. ELO2 encodes a fatty acid elongase integral to the elongation of fatty acids up to 24 carbons in length, a process that is upstream of sphingolipid biosynthesis and inherently also impacts cell wall integrity [35,36]. SLG1 encodes a sensor–transducer in the plasma membrane integral to the transduction of stresses detected at the cell wall, the organization of the actin cytoskeleton, mitophagy, pexophagy, and cell wall organization [37,38]. 

### 3.3. Metabolomic Analysis Reveals Increased Membrane Permeability and Increased Intracellular Phenolic Acid Accumulation Induced by DEA

To further investigate the genetic mechanisms identified above, changes in the relative abundance of intra- and extracellular metabolites of the most DEA-sensitive elo2∆ strain and its BY4741 parental strain were determined in the presence and absence of DEA over time using gas chromatography–mass spectrometry (GC–MS). Four cell populations (two conditions for two strains) were treated with an IC_20_ concentration of DEA or vehicle control at four different time points throughout the exponential growth phase (T0, immediately after the treatment; T1, 1 h after the treatment; T2, 2 h after the treatment; T3, 3 h after the treatment; and T4, 4 h after the treatment). In BY4741, 91 intracellular and 92 extracellular metabolites were accurately identified based on their chromatographic retention times and mass spectra, and similarly in elo2∆ cultures, 92 intracellular and 92 extracellular metabolites were accurately identified (Tables S1 and S2). 

**Figure 3:**
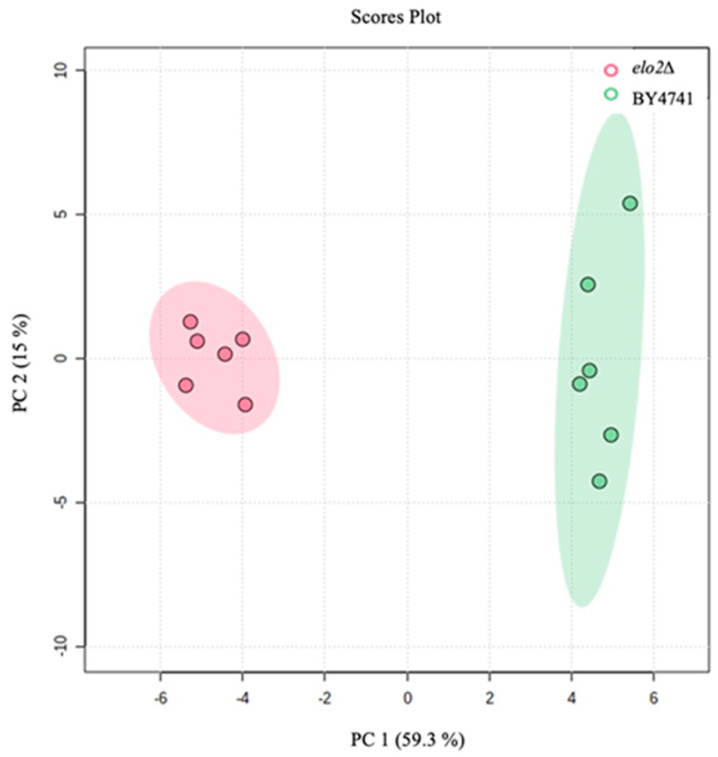
Two-dimensional projection of principal component analysis (PCA) showing the variation in intracellular metabolite profiles of two *Saccharomyces cerevisiae* strains (BY4741 parental strain and elo2∆ deletion). Samples were harvested at mid-log phase immediately after the addition of disalt of epipyrone A (DEA) or medium (time zero, T0).

To evaluate these metabolite profiles at T0, a principal component analysis (PCA) was conducted to reduce the complexity of these data. With the PC1 and PC2 axes explaining ~75% of the variance, the metabolite profiles were clearly distinct for BY4741 and elo2∆ (Figure 3). Notably, the clustering was independent of the DEA treatment, which was expected at T0. Therefore, the metabolite profiles confirm there were no significant differences within the replicates of strains or conditions at this time point, and moreover, these data reflect fundamental metabolic differences between the BY4741 and elo2∆ strains. 

**Figure 4:**
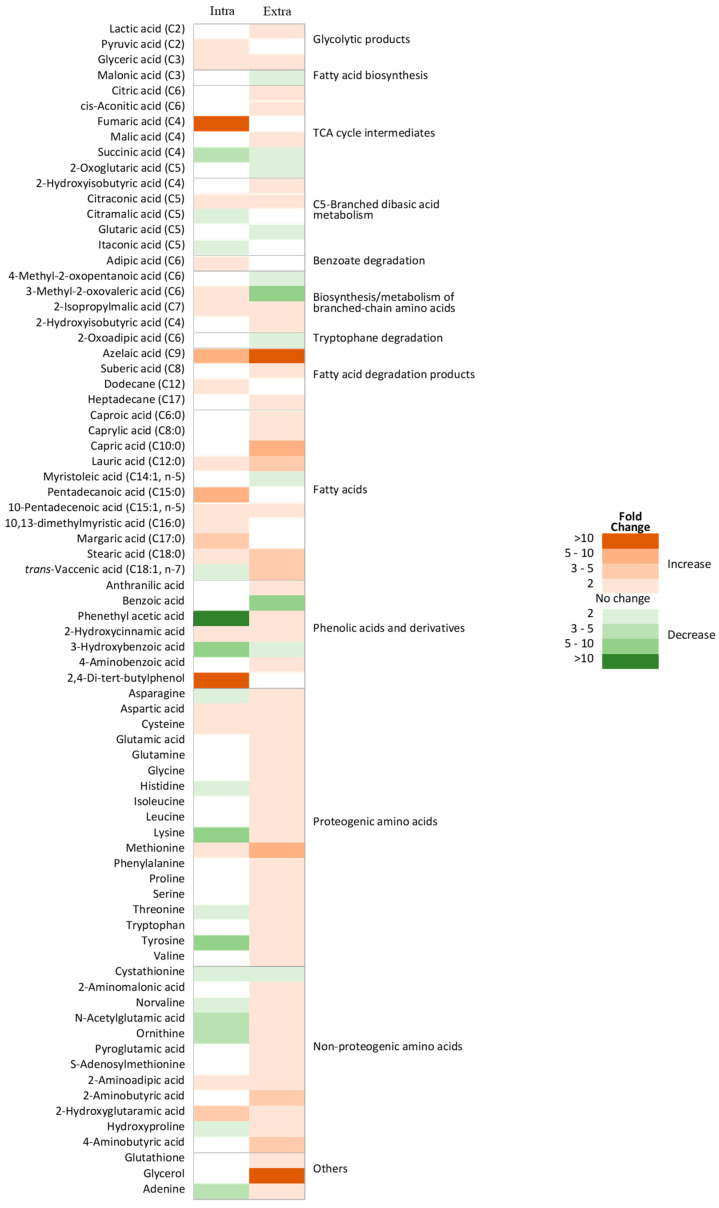
Heat map showing differences in intra- and extracellular metabolome of *Saccharomyces cerevisiae* elo2∆ and its parental strain (BY4741) immediately after the addition of disalt of epipyrone A (T0). Intra, intracellular; Extra, extracellular.

To investigate the metabolic effects of DEA, changes in the metabolic phenotypes were compared between BY4741 and elo2∆. At T0, the levels of 39 intracellular and 70 extracellular metabolites were significantly different (*p* < 0.05) between the strains (Figure 4). Among these, 22 intracellular and 56 extracellular metabolites were significantly increased in elo2∆ compared to BY4741. As expected, given the function of ELO2 in elongation of fatty acids up to 24 carbons, and considering that this mutant presents very pronounced differences in lipid composition [39], the level of most of fatty acids detected (C6 to C18) showed different (increased) levels in elo2∆ cells. Interestingly, trans-vaccenic acid (C18:1, n-7) was decreased intracellularly and increased extracellularly. Myristoleic acid (C14:1, n-5) showed a decreased extracellular level unlike other fatty acids. The fatty acid degradation products azelaic and suberic acids, as well as the alkanes dodecane and heptadecane, along with glycerol, were detected at significantly higher levels in the mutant samples as a clear indication of impaired biosynthesis of complex lipids. For example, azelaic acid showed 5- and 10-fold increases in intracellular and extracellular levels, respectively. Higher levels of all detected proteogenic amino acids, in addition to adenine and 4-aminobenzoic acid, were detected in the extracellular samples of elo2∆ mutant when compared to the parental strain. However, most of these metabolites were not detected and/or did not show significantly lower levels intracellularly. The exceptions were aspartic acid, cysteine, and methionine, the levels of which all were increased at intra- and extracellular locations in elo2∆ compared to BY4741. Interestingly, the metabolism of these three amino acids has a common product, serine, which is a key precursor for the biosynthesis of sphingolipids [40,41]. 

**Figure 5:**
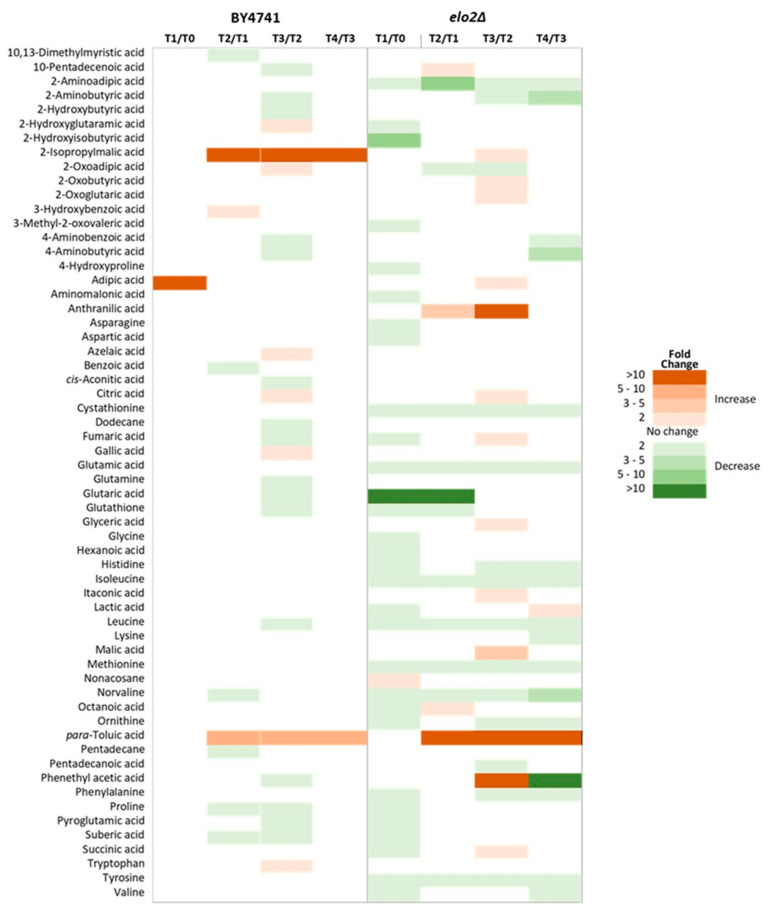
Heat map showing changes in intracellular metabolite levels of *Saccharomyces cerevisiae* cells (elo2∆ and its parental, BY4741) in response to disalt of epipyrone A (DEA). Samples were taken at T0 (immediately after the addition of DEA), then every 60 min for 4 h (T1-4).

**Figure 6:**
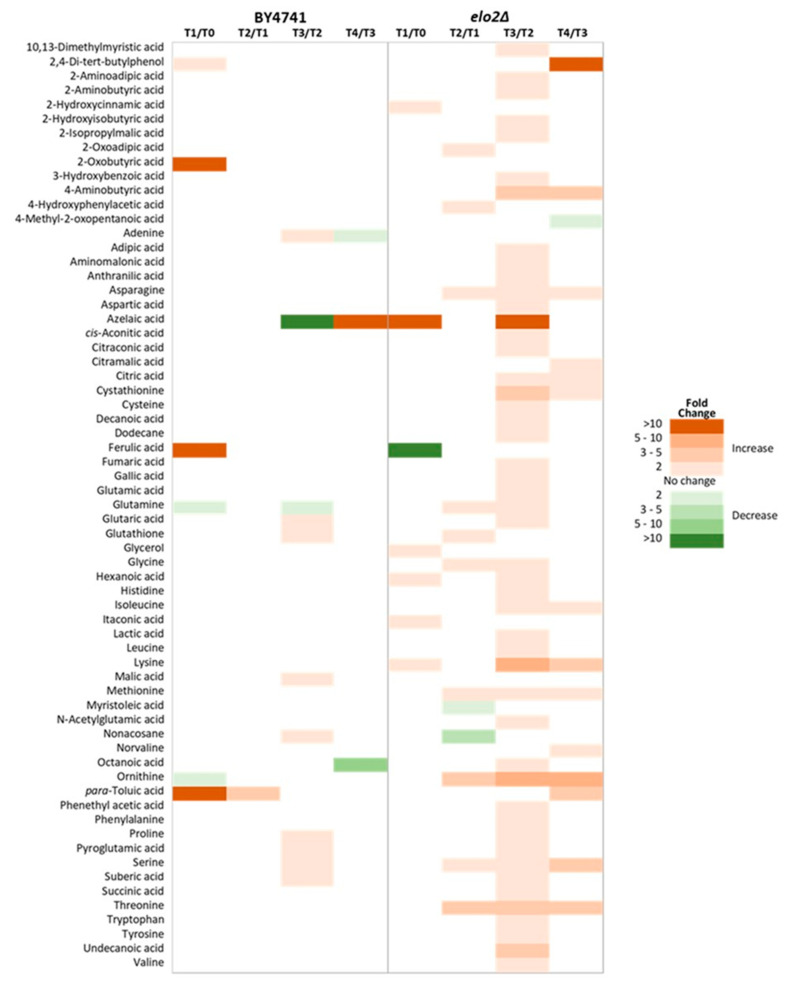
Heat map showing changes in extracellular metabolite levels of *Saccharomyces cerevisiae* cells (elo2∆ and its parental, BY4741) in response to disalt of epipyrone A (DEA). Samples were taken at T0 (immediately after the addition of DEA), then every 60 min for 4 h (T1-4).

To investigate how elo2∆ and its parental strain respond to DEA over time, cells were incubated in the presence and absence of the antifungal compound for four hours. Throughout the incubation, 49 intracellular and 63 extracellular metabolites were significantly altered in elo2∆, while only 31 intracellular and 22 extracellular metabolites changed in its parental strain (Figure 5 and Figure 6). In BY4741, DEA resulted in an initial 5-fold increase in adipic acid within the first hour of incubation, followed by a significant increase in the levels of 2-isopropylmalic and p-toluic acids (Figure 5). Adipic and p-toluic acids are common degradation products of aromatic compounds [42]. Consistently, other phenolic acids and derivatives such as 3-hydroxybenzoic and gallic acids, as well as tryptophan and several other organic acids, also exhibited increased intracellular levels. No pronounced and/or prolonged reductions in intracellular metabolites were observed over the incubation period, except the significant 2-fold decrease in proline and suberic acid levels between T2 and T3. Only the level of 2-isopropylmalic and p-toluic acid increased significantly at T4, while the levels of the other metabolites stabilised (Figure 5), which indicates that these two metabolites may be intrinsically involved with DEA metabolism. 

In contrast, the DEA-treated elo2∆ did not show an initial decrease in the level of adipic acid or a significant/prolonged increase in the intracellular levels of 2-isopropylmalic acid (Figure 5). However, the level of p-toluic acid, a known toxic compound [43], showed a continuous increase intracellularly over time and even more pronounced than in the parental strain. Considering that the parental strain also showed a parallel significant increase in p-toluic extracellularly in T1 and T2, suggesting an active secretion of this toxic phenolic acid, this indicates that elo2∆ mutation may have affected the ability of the yeast cell to efficiently keep the excess of p-toluic acid outside the cell. Therefore, increased levels of p-toluic acid could be a downstream cellular effect of DEA. Distinct to elo2∆, the levels of anthranilic and phenylethyl acetic acid were also increased intracellularly; anthranilic acid was increased at T2 until T3, while phenyl ethyl acetic acid was increased at T3. With the apparent diminished ability to keep the excess of p-toluic acid outside the cell, this compound is likely to have induced further biochemical transformations inside elo2∆ cells, explaining the increased levels of other aromatic compounds, probably derived from p-toluic acid. Contrary to the parental strain, elo2∆ also showed a significant decrease in the level of several intracellular metabolites in the first hour in the presence of DEA, such as several proteogenic amino acids, 2-hydroxyisobutyric, and glutaric acids, which could suggest an increased secretion of these metabolites. The most pronounced decrease was glutaric acid with a 10-fold reduction compared to T0. Additionally, decreased levels of several intracellular metabolites were perdured for the whole or majority of the incubation period, such as 2-aminoadipic acid, cystathionine, and many proteogenic amino acids. 

Changes in the level of extracellular metabolites of both elo2∆ mutant and its parental strain over time in response to DEA were compared (Figure 6). Overall, more secretion of metabolites to the extracellular medium was observed than uptake. The changes in the level of extracellular metabolite in response to DEA were more significant in the deletion mutant, especially at T3 (Figure 6). The mechanism behind the reasons a cell secretes intracellular metabolites to the extracellular medium is complex and poorly studied to date, and these include intracellular metabolic overflow, metabolite toxicity, and changes in cell membrane permeability [44,45]. Considering the nature of elo2∆ mutation associated with fatty acid elongation and in sphingolipid biosynthesis [35,36], and the reported pronounced differences in the lipid composition of this mutant [39], it is very likely that DEA affects the cell membrane permeability, with which the parental strain seems to be able to cope better than the deletion strain, emphasizing the important role of long-chain fatty acids and sphingolipids in the cell membranes to protect the fungal cells from DEA. Interestingly, this change in cell permeability induced by elo2∆ mutation negatively affected the ability of the cell to keep the excess of toxic p-toluic extracellularly (Figure 6). 

### 3.4. Fluorescence Microscopy Confirms Interruption of Sphingolipid Biosynthesis by DEA

**Figure 7:**
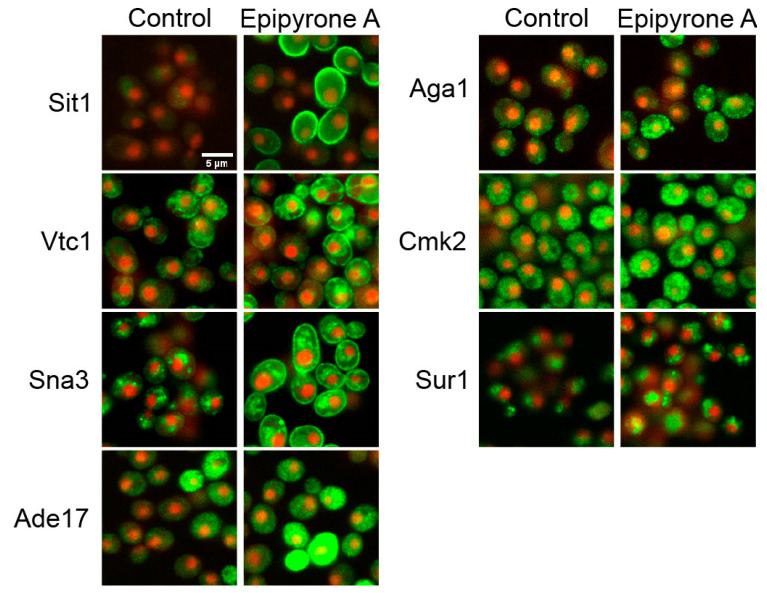
Disalt of epipyrone A (DEA) alters the abundance or localization of seven GFP-tagged proteins. Cells were grown for 8 h in absence or presence of 25.5 µg/mL DEA (IC_20_) in synthetic complete broth, then imaged using an Incell analyser 6500HS (General Electric). For reference, nuclei were distinguished with RFP. These proteins include Sit1(YEL065W); Vtc1 (YER072W); Sna3 (YJL151C); Ade17 (YMR120C); Aga1 (YNR044W); Cmk2 (YOL016C); and Sur1 (YPL057C).

**Figure 8:**
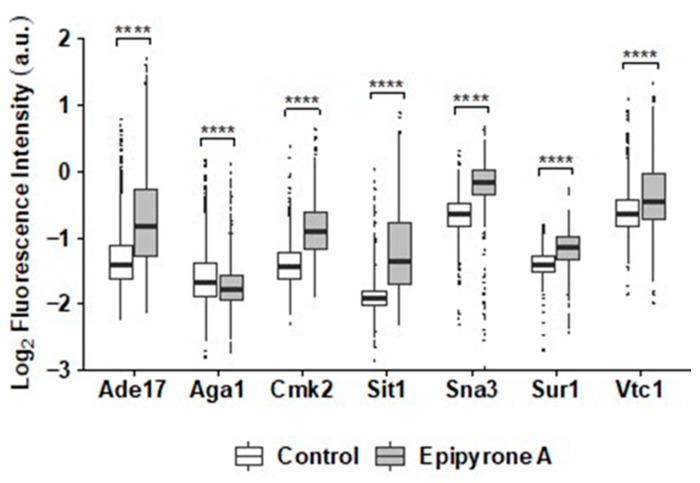
Quantitative comparison of GFP-tagged protein intensity in presence and absence (control) of disalt of epipyrone A (DEA). Cells were grown for 8 h in absence or presence of 25.5 µg/mL DEA (IC_20_) in synthetic complete broth, then imaged using an Incell analyser 6500HS (General Electric). These proteins include Ade17 (YMR120C); Aga1 (YNR044W); Cmk2 (YOL016C); Sit1(YEL065W); Sna3 (YJL151C); Sur1 (YPL057C); and Vtc1 (YER072W). **** represents *p* < 0.0001.

To investigate the mechanism of DEA at the protein level, we measured changes in the abundance and localization of 4300 proteins in a GFP-tagged S. cerevisiae library, where each strain contains C-terminus tagged GFP under the control of the endogenous promoter fused to a different protein [30]. Seven showed significant changes regarding their localization (Figure 7) and/or intensity of the GFP-tagged proteins in response to DEA (Figure 8). Sit1-GFP and Sna3-GFP changed their localization from the vacuole to the cell periphery. These are proteins involved in ferrioxamine B permease [46] and in the efficient sorting of proteins in multivesicular bodies (MVBs) to the vacuole [47], respectively. In addition to these, four other GFP-tagged proteins showed increased intensity with DEA treatment. These were Vtc1, the subunit of the vacuolar transporter chaperone (VTC) complex [48]; Ade17, an enzyme involved in the de novo biosynthesis of purine [49]; Cmk2, a calmodulin-dependent protein kinase [50]; and Sur1, a subunit for mannosyl-inositol phosphoryl ceramide (MIPC) synthesis in the sphingolipid biosynthesis pathway [51,52]. The surface glycoprotein agglutinin Aga1 [53] showed a decrease in its abundance in response to DEA. Aga1 and Sit1showed significant decreases in the intensity of the RFP-tagged proteins, while Cmk2, Sur1 and Vtc1 showed significant increases.

### 3.5. Thin Layer Chromatography Confirms the Effect of DEA on Lipid Metabolism

**Figure 9:**
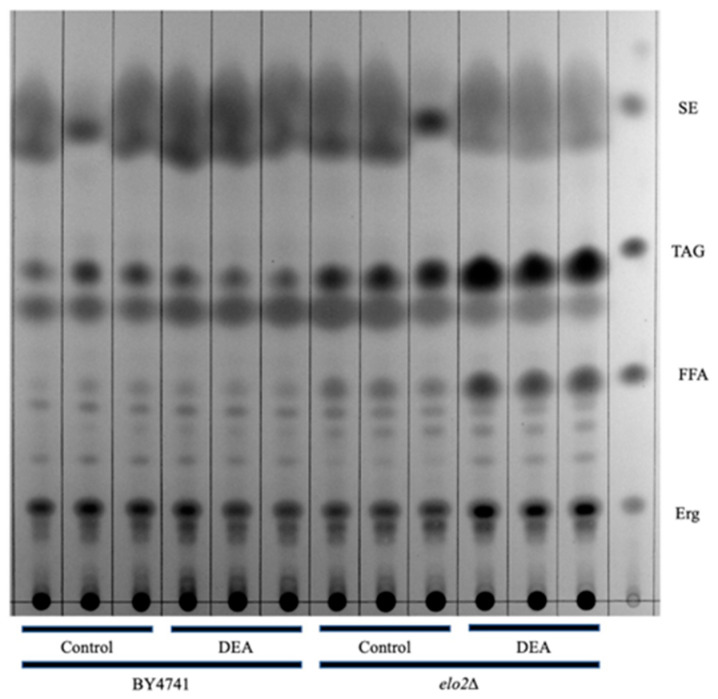
Thin-layer chromatography (TLC) reveals accumulation of free fatty acids (FFA) and triacyl glycerides (TAG) in disalt of epipyrone A (DEA)-treated cells. Cells were grown for 8 h in presence or absence of 25 ug/mL DEA. Lipids were extracted, separated using hexane/petroleum, ether/diethyl, and ether/acetic acid (50:20:5:1, *v*/*v*/*v*/*v*) as solvent and stained with iodine vapor. FFA, free fatty acids; Erg, ergosterol; TAG; triacylglycerols; SE, steryl esters.

The hypersensitivity of ELO2-deficient yeast to DEA suggests lipid metabolism and membrane composition may play a major role in the bioactivity. We thus used thin-layer chromatography (TLC) to compare the levels of major lipid groups in BY4741 and elo2∆ cells in the presence and absence of DEA at 25.5 μg/mL (Figure 9). While free fatty acid (FFA) levels were not increased in DEA-treated BY4741 compared to untreated BY4741, the FFA levels were increased at least 2-fold in DEA-treated elo2∆ compared to untreated elo2∆. Consistent with the literature and metabolomics results [39], the FFA levels were increased in untreated elo2∆ compared to untreated BY4741 (Figure 4). To a lesser extent, the levels of triacyl glycerides (TAG) also increased in DEA-treated elo2∆ compared to untreated elo2∆, and their levels appeared to be slightly increased in untreated elo2∆ compared to untreated BY4741 (Figure 4). In contrast, the ergosterol levels were not altered between any strain or condition. These results provide a mechanistic basis for the hypersensitivity of elo2∆ to DEA, wherein FFA levels accumulate in DEA-treated and untreated elo2∆, demonstrating an impaired incorporation of these fatty acids into complex lipid molecules, which is worsened by DEA. Defective elongation of fatty acids and their subsequent incorporation into complex lipid molecules such as sphingolipids would result in the accumulation of short- and medium-chain fatty acids in the cells, as pointed out by metabolomics, which could also result in an increased biosynthesis of TGAs containing short- and medium-chain fatty acids (Figure 4).

## 4. Discussion

The most interesting result in this study is the finding that the potential antifungal mode of action of DEA involves the disruption of sphingolipid/fatty acid biosynthesis in *S. cerevisiae*. Through chemical-genetic profiling, we identified that the elo2∆ mutant strain showed hypersensitivity towards the IC_20_ of DEA (Figure 2). Fatty acid elongase is a membrane-bound enzyme that plays a crucial role in the elongation of fatty acid, which leads to the synthesis of very long-chain fatty acids (VLCFAs) of C20:0-C24:0 [36,54]. VLCFAs ultimately participate in complex sphingolipid formation including inositol-phosphoceramide (IPC), mannose-inositol-phosphoceramide (MIPC), and mannose-(inositol-P)2-ceramide (M[IP]2C) [36,55]. Sphingolipids with VLCFAs of C20:0-24:0 are known to constitute only a minor part of the yeast cytoplasmic membrane, while the major constituents are the ones with C26:0 that are elongated by Elo3 [54,56]. Nonetheless, elo2∆ mutation is known to produce pronounced changes in the lipid composition of yeast cells [39]. Changes in the composition of sphingolipids and other lipids of the yeast membrane, which is well-known to alter the properties of the membrane [48], may have resulted in varied responses against the stressor, including increased overall membrane permeability, as evidenced by our metabolomics study. Given that elo2∆ previously showed reduced levels of sphingolipids, as well as increased MIPCs with shorter fatty acids [55], the hypersensitivity observed in this research in elo2∆ indicates that long-chain fatty acids and sphingolipids that are minor constitutes (C20:0-C24:0) in the yeast membrane may be providing some resistance to the yeast against the antifungal compound, influencing inclusively the ability of the cell to excrete an excess of *p*-toluic acid, a potential toxic catabolic product of DEA. 

The increased abundance of Sur1, a subunit of MIPC synthase that is involved in IPC to MIPC synthesis through mannosylation [57,58,59], indicates a possible accumulation or overexpression of Sur1 due to the interruption of sphingolipid biosynthesis. DEA may have reduced the substrate IPC, which may lead to deficiency in other complex sphingolipids, such as MIPC and M(IP)2C, that are present in the yeast membrane. The lack of ability to transfer mannose to IPC for the synthesis of MIPC is the common characteristic of sur1∆ mutant strains, as well [51]. It was previously reported that *Saccharomyces pombe* with MIPC deficiency exhibit accumulation of substrate-dependent permease Aat1 in the plasma membrane [60]. It was speculated that the protein was transported to the yeast membrane to acquire nutrients, but failed to be internalized and sorted through the multivesicular body (MVB) pathway for degradation [60]. Ubiquitination and MIPC mannose residues were thought to play a crucial role in this [60]. On the other hand, the changes in the localisation of the Sit1 and Sna3 proteins of *S. cerevisiae* from the vacuole to cell periphery in the presence of DEA may be due to the fact that these two proteins are known to require ubiquitination to be internalised through the MVB sorting pathway [61,62]. This suggests the possible disruption of their internalization through MVB sorting by the antifungal compound, which may have been caused by a reduced level of MIPC, corroborating with the observed increased abundance of Sur1 in the presence of DEA due to, possibly, the lack of IPC substrates that led to the reduced synthesis of MIPC. 

*S. cerevisiae* with the elo2∆ mutation previously showed increased sensitivity against amphotericin B (AmB), another polyene-class antifungal drug [63], as well. Polyene-class antifungal drugs are well-known to bind ergosterol in the fungal membrane[(1,2]. However, we did not observe any changes in the ergosterol level in either elo2∆ or its parental strain in the presence and absence of DEA through TLC (Figure 9). Only a minor difference in the structure of a chemical compound was shown to result in drastic changes in its biological activity [23,24] and/or additional modes of action [13,25,26]. DEA possesses a polyene chain, however, in a linear structure. This is unlike other drugs of the polyene family, which possesses a cyclic structure with an associated polyol moiety that is necessary for membrane pore formation and the efflux of cellular components. This likely indicates different antifungal modes of action of DEA compared to the other polyene class of antifungal drugs, including the strong possibility of a distinct binding target within the cell membrane. 

## 5. Conclusions

Fungal sphingolipids are known to participate in several crucial roles in fungal cell physiology, such as signal transduction, endocytosis of membrane protein, and stress responses, in addition to being structurally different from the sphingolipids found in mammalian cells or even completely absent altogether in mammalian systems [3,64]. Therefore, they are emerging as potential targets for future antifungal drugs. Considering all evidence observed in this research through chemical-genetics, metabolomic profiling using GC–MS, fluorescence microscopy, and TLC, it is possible DEA affects fatty acid elongation and sphingolipid biosynthesis with consequent changes in cell membrane composition and permeability. Moreover, our metabolomic data showed that DEA exposure induces increased production of *p*-toluic acid intracellularly, which is toxic to the cell at elevated concentrations [43]. 

## Data Availability

Available from the corresponding authors upon reasonable request.

## Supplementary Materials

The following supporting information can be downloaded at https://www.mdpi.com/article/10.3390/jof10090597/s1: Table S1: the Incell analyser 6500HS (General Electric) settings used for the green-fluorescent library screening; Table S2: the list of intra- and extra-cellular metabolites of *Saccharomyces cerevisiae* BY4741 parental strains that were accurately identified with the in-house MS library; and Table S3: the list of intra- and extra-cellular metabolites of *Saccharomyces cerevisiae* elo2Δ deletion strains that were accurately identified with the in-house MS library.
