# Supplementary material for: Antifungal Activity of Disalt of Epipyrone A from Epicoccum nigrum Likely via Disrupted Fatty Acid Elongation and Sphingolipid Biosynthesis
Source: J Fungi (Basel). 2024 Aug 23;10(9):597. doi: 10.3390/jof10090597 (PMC11433475; doi:10.3390/jof10090597)
Supplement: Supplementary file 1 [file jof-10-00597-s001.zip › Table S1.pdf]

Table S1. The Incell analyser 6500HS (General Electric) settings used for the green-fluorescent library screening.

Parameter	Setting
Objective	Nikon 60X/0.95, Plan Apo, Corr Collar 0.11-0.23, CFI/60 Lambda
Binding	1 x 1
Wavelengths	488 nm and 561 nm
Exposure	500 ms and 500 ms
AF Offset	2.5
Image mode	2-D
Aperture	1.56 AU and Open Aperture
