# Supplementary material for: Antifungal Activity of Disalt of Epipyrone A from Epicoccum nigrum Likely via Disrupted Fatty Acid Elongation and Sphingolipid Biosynthesis
Source: J Fungi (Basel). 2024 Aug 23;10(9):597. doi: 10.3390/jof10090597 (PMC11433475; doi:10.3390/jof10090597)
Supplement: Supplementary file 1 [file jof-10-00597-s001.zip › Table S2.pdf]

Table S2. The list of intra-and extra-cellular metabolites of *Saccharomyces cerevisiae* BY4741 parental strain that were accurately identified with the in-house MS library.

Classification of metabolites	Intra	Extra	Metabolites
**Proteinogenic amino acids**	18	18	Cysteine, aspartic acid, glutamic acid, phenylalanine, glycine, histidine, isoleucine, lysine, leucine, methionine, asparagine, proline, glutamine, serine, threonine, valine, tryptophan, tyrosine
**Non-proteinogenic amino acids**	8	8	2-aminoadipic acid, 2-aminobutyric acid, 4-aminobutyric acid, cystathionine, N-acetylglutamic acid, norvaline, ornithine, pyroglutamic acid
**TCA cycle intermediates**	6	6	2-oxoglutaric acid, cis-aconitic acid, citric acid, fumaric acid, malic acid, succinic acid
**Fatty acids**	20	20	10,13-dimethyltetradecanoic acid, 10-pentadecenoic acid, 2-oxoadipic acid, 2-oxobutyric acid, adipic acid, decanoic acid, dodecane, dodecanoic acid, hexanoic acid, margaric acid, myristic acid, myristoleic acid, octanoic acid, palmitelaidic acid, pentadecane, pentadecanoic acid, stearic acid, suberic acid, trans-vaccenic acid, undecanoic acid
**Others**	39	40	Dimethyl aminomalonic acid, 2,4-di-tert-butylphenol, 2-hydroxybutyric acid, 2-hydroxycinnamic acid, 2-hydroxyglutaramic acid, 2-hydroxyisobutyric acid, 2-isopropylmalic acid, 3-acetoxy-3-hydroxy-2-methylpropionic acid, 3-hydroxybenzoic acid, 3-methyl-2-oxovaleric acid, 4-aminobenzoic acid, 4-hydroxyphenylacetic acid, 4-methyl-2-oxopentanoic acid, anthranilic acid, azelaic acid, benzoic acid, caffeine, cis-4-hydroxyproline, citraconic acid, citramalic acid, creatinine, ferulic acid[a], gallic acid, glutaric acid, glutathione, glyceric acid, glyoxylic acid, heptadecane, itaconic acid, lactic acid, malonic acid, NADP/NADPH, nicotinamide, nonacosane, para-toluic acid, phenethyl acetate, pimelic acid, pyruvic acid, s-adenosylmethionine, tricosane
**Total**	91	92	

Intra: Intracellular metabolites identified; Extra: Extracellular metabolites identified. [a]Metabolites that were only found extracellularly; [b]Metabolites that were only found intracellularly.
